# Surgery remember^@^: an innovation to reduce surgical cancellations

**DOI:** 10.1590/0100-6991e-20213206

**Published:** 2021-12-17

**Authors:** DANIELLY ACIOLI GALVÃO DE SOUZA, IRAMI ARAÚJO-FILHO, ERIC LUCAS DOS SANTOS CABRAL, RICARDO PIRES DE SOUZA, ALEXANDRE GUILHERME RODRIGUES VARELLA, ERIKA MARIA ARAÚJO BARBOSA DE SENA, AMÁLIA CINTHIA MENESES RÊGO, BRUNA LUIZA DE BARROS MELO, JOÃO FLORÊNCIO DA COSTA-JUNIOR, FRANCISCO IROCHIMA PINHEIRO

**Affiliations:** 1 - UFRN, Programa de Pós Graduação em Gestão e Inovação em Saúde - Natal - RN - Brasil; 2 - Hospital Universitário Alberto Antunes - HUPAA, Centro Cirúrgico - Maceió - AL - Brasil; 3 - Laureate Universities - Universidade Potiguar, Programa de Pós Graduação em Biotecnologia - Natal - RN - Brasil; 4 - Universidade Federal do Rio Grande do Norte, Departamento de Cirúrgia Experimental - Natal - RN - Brasil; 5 - Universidade Federal do Rio Grande do Norte, Programa de Pós Graduação em Engenharia de Produção - Natal - RN - Brasil; 6 - Universidade Federal do Rio Grande do Norte, Departamento de Engenharia de Produção - Natal - RN - Brasil; 7 - Universidade Federal do Alagoas, Instituto de Química e Biologia - RENORBIO/ Ponto focal UFAL - Maceió - AL - Brasil; 8 - Universidade Federal do Rio Grande do Norte, Departamento de Ciências Administrativas - Natal - RN - Brasil

**Keywords:** Smartphone, Reminder Systems, Software, Surgery Department, Hospital, Absenteeism, Smartphone, Sistemas de Registro de Ordens Médicas, Software, Centro Cirúrgico Hospitalar, Absenteísmo

## Abstract

The use of mobile phones has dramatically increased all over the world. Such revolution in the communication amongst individuals has a great impact in patient care, supporting their self-management and promoting shared responsibility with health services. Given that improved communication facilitates compliance with scheduled procedures and reduces surgical cancellations, the current work aims to develop a communication tool named Surgery Remember^@^ to mitigate surgical suspensions due to patient absenteeism. The present article is a study of technological production divided into four chapters: literature review; analysis of the hospital administrative profile; software development; and process mapping for software implementation. Taking into account that in the last three years the problem of absenteeism was the main cause of institutional surgical cancellations; the development of Surgery Remember^@^ endeavours to reduce surgery cancellations, improving efficiency and reducing costs. It is known that sending messages three days before the surgical procedure makes it possible to replace patients in the event of cancellations, optimizing the human and material resources in the operating room. The confirmation of the pre-aesthetic consultation is also positive, for it allows the verification of perioperative assistance improvement. Hence, besides being viable and easy to implement, the software developed allows the addition of other features based on user requirements, proving to be an asset to reduce surgery cancellations.

## RESUMO

O uso de telefones celulares aumentou dramaticamente em todo o mundo. Essa revolução na comunicação entre os indivíduos tem impacto no cuidado ao paciente, apoiando sua autogestão e promovendo a responsabilidade compartilhada com os serviços de saúde. Tendo em vista que a melhoria da comunicação facilita o cumprimento dos procedimentos programados e reduz os cancelamentos cirúrgicos, o presente trabalho tem como objetivo desenvolver uma ferramenta de comunicação denominada Surgery Remember^@^ para mitigar as suspensões cirúrgicas por absenteísmo do paciente. O presente artigo é um estudo da produção tecnológica dividido em quatro capítulos: revisão de literatura; análise do perfil administrativo do hospital; desenvolvimento de software; e mapeamento de processos para implementação de software. Tendo em conta que nos últimos três anos o problema do absenteísmo foi a principal causa de cancelamentos cirúrgicos institucionais o desenvolvimento do Surgery Remember^@^ se apresenta como esforço para reduzir os cancelamentos de cirurgias, melhorando a eficiência e reduzindo custos. Sabe-se que o envio de mensagens três dias antes do procedimento cirúrgico possibilita a reposição de pacientes em caso de cancelamentos, otimizando os recursos humanos e materiais da sala cirúrgica. A confirmação da consulta pré-anestésica também é positiva, pois permite verificar a melhora da assistência perioperatória. Assim, além de viável e de fácil implementação, o software desenvolvido permite agregar outros recursos de acordo com a necessidade do usuário, mostrando-se uma ferramenta para redução de cancelamentos cirúrgicos.

## INTRODUCTION

The use of mobile phones has increased dramatically, with nearly 7 billion users worldwide, of which 89% live in developing countries. Such transformation in the communication process amongst people provides great potential for improvements in patient care, supporting self-management by promoting shared responsibility with health services^1^ ^,^ ^2^.

According to the World Health Organization, the practice of supporting medical health with communication technology and mobile devices is defined as Mobile Health (mHealth)^3^. Due to its mobility characteristics, diversity of functionality, instant access, and connectivity, mHealth influences the attitudes and behaviours of patients, besides promoting the exchange of information between patients and health professionals^4^. 

The use of mHealth apps is effective in monitoring users with chronic diseases such as diabetes, asthma and high blood pressure as well as supporting weight loss and the treatment of smoking addiction, besides sending reminders for consultation and surgical procedures^4^ ^,^ ^5^. 

The insertion of short text (SMS) or multimedia (MMS) services in the healthcare environment appears to be an innovative approach for inducing patient involvement, behaviour change and treatment adherence^6^ ^,^ ^7^.

The use of such technologies in healthcare has as main representative the WhatsApp messaging application, due to its functionality that allows the possibility of immediate response, besides the sharing of text, video, voice, and image messages over the internet on a secure network platform^8^ ^,^ ^9^.

Previous research has established that improving patient communication with healthcare services facilitates compliance with scheduled procedures and reduces surgical cancellations^10^ ^,^ ^11^. Usually, preoperative contact with the patient is carried out by telephone; however, this method has some disadvantages such as restricted appropriate times and the comparatively cumbersome and time-consuming process of making calls. Meanwhile, automated message reminders allow for greater availability and seamless information access, being efficient and less invasive in people’s daily lives compared to telephone calls^5^. 

Based on the premise that patient absenteeism is the main cause of surgical cancellation in the last three years in the public university hospital analysed by the current authors, the present research aimed to develop a communication tool - Surgery Remember^@^ - to reduce surgical cancellations through seamless communication with patients.

## METHOD

The current work is a technological production study, to develop a message management tool called Surgery Remember^@^, aimed at mitigating surgical cancellations due to patient absenteeism at the Professor Alberto Antunes - HUPAA University Hospital located in the city of Maceió in Alagoas, Brazil.

The research resulted in a solution designed to reduce absenteeism of patients with previously scheduled surgery, satisfying the criteria of feasibility, reliability, efficiency of the inventions as well as the expected cost-benefit ratio^12^. The system had the following development phases: i) data collection; ii) modelling; iii) coding; iv) development; and v) future validation. Moreover, a bibliographic survey was carried out in the PubMed, Scopus, and Web of Science databases between 2015 to 2020; using the following descriptors: “smartphone”; “reminder system”; “software”; “surgery department hospital”; “absenteeism”.

The key question to guide the research was: “What has been developed to date in scientific research in the healthcare field related to the use of instant messaging as a reminder of attendance to patients with scheduled surgery?” Based on the search strategies mentioned, twenty-one articles were potentially relevant, of which twelve were selected to compose the sample, as they were directly related to the research question and objective.

**Figure 1:**
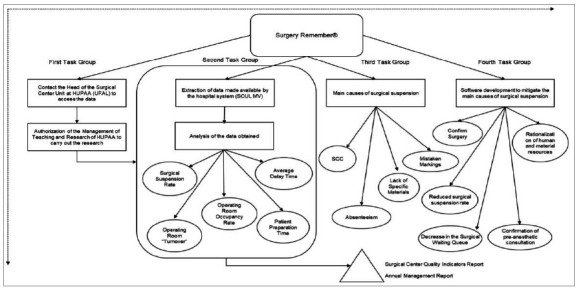
Methodological approach.

The selected article review was carried out initially by reading the titles, followed by the analysis of the abstracts and subsequent appreciation of the full texts and verification of compatibility with the purpose of the present study. The methodological process is depicted in Figure 1, presenting the systematization subdivided into the following groups; i) tasks; ii) problem analysis; and iii) strategy elaboration for the Surgery Remember^@^ software. 

In the first stage, the Head of the Surgical Centre and Teaching and Research Management Unit at HUPAA was contacted for consent. In the second stage, the data, and indicators of the operating room, made available by Soul MV^®^, were analysed, highlighting those of greater relevance. The third stage emphasized the rate of surgical cancellation and its main causes: absenteeism, patients without clinical conditions, lack of materials, and wrong scheduling. Finally, the Surgery Remember^@^ software was developed, aimed at reducing the main causes of surgical cancellation.

Regarding the analysis of the indicator for cancellation causes in the period from 2017 to 2019, the Pareto principle was utilized, facilitating the investigation and communication of information, by prioritizing the problems that accounted for most of the cancellations^12^.

## RESULTS

The integrative review included 11 articles published in English and one in Spanish. Most of the analysed articles reported the use of messages to monitor patients with chronic diseases, smoking cessation, anxiety control, use of medications, amongst others.

With regards to the theme of absenteeism, none of the researched articles addressed the use of instant messages aimed at the surgical patient. Thus, the importance of further exploring this theme focused on this patients’ profile is emphasized.

At the current studied hospital, the analysis of the causes of cancellation of 1,574 operations out of a total of 4,614 performed in 2019 represented a cancellation rate of around 25.44%. Amongst the main causes, the following are highlighted: absence of the patient (19%), lack of clinical conditions (14%) and wrongly scheduled operations (8%).

The above data bears similarity to the 2018 data, when patients’ absence corresponded to 22% of the total surgical cancellations: and the lack of clinical conditions, 16%. In 2017, the patient’s non-attendance reached the most expressive amount, peaking at 27%.

**Figure 2:**
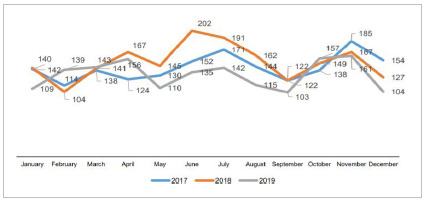
Monthly quantity of operations cancelled in the years of 2017, 2018 and 2019.

After the evaluation based on the Pareto principle, the most significant cancellation causes were established, which led to the prioritization of causes. The impact of patient absenteeism on the elevation of the surgical cancellation rates over these three years is rather evident. Despite its reduction in the period under study, it remains considerably high. The graphical representations of the cancellation rate in the years 2017 - 2019 are shown in Figure 2.

It is also noticed that the year 2018 stands out in the number of cancelled operations, representing a total of 1,819 cancellation; whilst the year 2019 revealed the lowest amount, 1,574.

**Figure 3:**
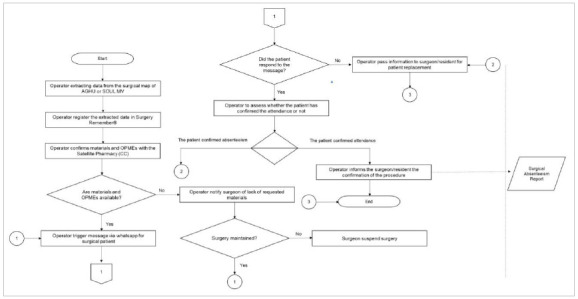
Surgery Remember ^@^ operational flowchart.

Each component of the system as well as an overview of its architecture will be described below. The flowchart shown in Figure 3, describes the software’s functionalities and its applicability in the hospital under study.

**Figure 4:**
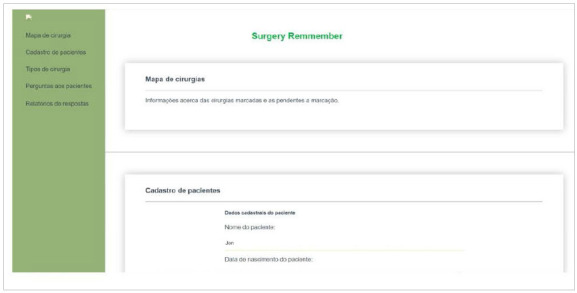
Login screen - Surgery Remember ^@^ .

The main screen is intended for user login and to register patients with operations scheduled at the hospital. The data of the surgical map of the hospital system is tabulated in an Excel® spreadsheet (Microsoft^®^/2016) and then transferred to the Surgery Remember^@^ software as shown in Figure 4.

**Figure 5:**
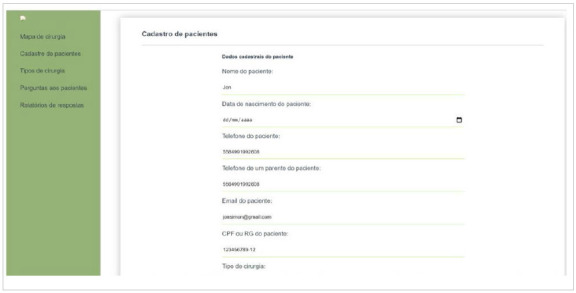
Home screen - Surgery Remember ^@^ .

Once login is concluded, the system directs the user to the preoperative form. Three days before the surgical procedure, attendance confirmation reminders are issued to previously registered patients (Figure 5).

**Figure 6:**
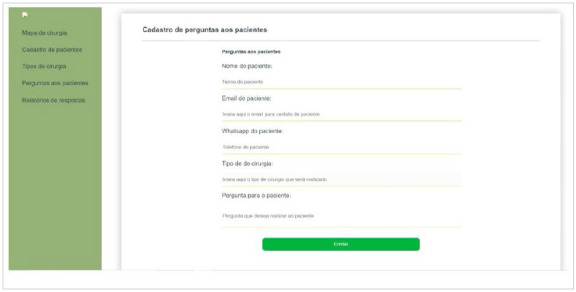
Screen for the surgical procedure confirmation message.

The professional responsible for surgical patients’ registrations will fill in the following data: patient name, phone number, patient record, type of procedure, surgeon in charged, and date of the operation. Once all information is completed, the record button is activated. Thereby, the system will send an instant message via WhatsApp to the registered contact phone, asking the patient to confirm their presence at the scheduled surgical procedure and pre-aesthetic consultation (Figure 6).

After sending instant messages to patients with a scheduled surgical procedure, the system will generate a report with the percentile of confirmed patients, pending confirmations and no-show confirmations.

It is worth emphasizing that measures to minimize surgical cancellations are highly recommended, resulting in the proper use of the operating room and the best use of available human resources as well as hospital supplies^14^. 

## DISCUSSION

Surgery cancellations are known to have a high impact on the organization and efficiency of hospitals. In the surgical scenario, cancellations of elective cases refer to operations that were not performed on the intended day. The cancellations of surgical procedures are a worldwide problem that result in inefficient usage of resources, which are generally scarce^15^. 

Surgical cancellations caused by the non-attendance can compromise patients’ health, due to lack of intervention in a timely manner; moreover, it decreases scheduling availability whilst increasing waiting time, impacting the prognosis^16^.

The use of mobile technologies has considerable potential to improve communication between hospital and patients, decreasing cancellations, through reminders and continuous education^17^. Thus, the use of instant messaging via smartphones can optimize efficiency in patient care^18^. Some authors^19^ have shown that the instant messaging service is an alternative form of communication between the doctor and the patient or his family, with significant advantages.

Reminder strategies are effective in decreasing the cancellation rate, although it is not clear which type of reminder is the most effective^16^ ^,^ ^20^. Several interventions with different reminder methods have been tested, concluding that there is a reduction in absenteeism rates, regardless of the used method^21^.

Intervention through previous telephone contact had a positive impact on surgical cancellations due to patients’ non-attendance in a university hospital in Alagoas, reducing cancellations caused by non-attendance by 50%^22^.

Research aimed at reducing absenteeism in paediatric consultations, found an effective decrease with the use of messages, reaching 8.5% of non-attendance compared to 11.9% by telephone contact, with about 83% of the participants preferring messages via WhatsApp^11^.

This type of instant messaging services has experienced in recent years an exponential growth in its popularity, becoming a very common form of communication, for personal and professional purposes, besides allowing fast data sharing and offering end-to-end encryption^23^. 

It is important to highlight that patients’ involvement has been a relevant component in the formulation of strategies to optimize healthcare services leading to positive results^24^ by encouraging patients to contact the institution in advance, informing the cancellation of the surgical procedure or scheduled appointment.

In a study on the causes of patient absenteeism, it was found that 29.7% was due to forgetfulness, 16% due to failed communication between the system and the patient and 57.8% was the result of new consultation requirements. It has been observed that reducing forgetfulness through reminders significantly decreases the frequency of non-attendance cases^16^. 

Amongst the current study’s limitations, it is important to highlight that some patients face difficulties in utilizing technological resources for guidance in relation to the surgical procedure. Furthermore, there may be legal and technological integration barriers between the hospital’s information management tools and the Surgery Remember^@^ software, limiting access to the surgical map information and, consequently, compromising the efficiency of the new process established. 

Lastly, it is expected that, despite some potential operational barriers, the use of the software will make it possible to confirm the surgical procedure and pre-aesthetic consultation, to reduce the waiting list, by replacing patients who intended to cancel or not to attend the surgical procedure. As a result, the rate of surgical cancellations and the utilization of human and material resources will be positively impacted.

## CONCLUSION

The communication tool Surgery Remember^@^ aims to reduce surgical cancellations due to patients’ absence. Messages sent three days before the surgical procedure will enable the replacement of patients in case of patient withdrawal, optimizing the use of human and material resources.

The confirmation of the pre-anesthetic consultation will be positively impacted on the verification of the perioperative assistance improvement. Therefore, Surgery Remember^@^, besides being viable and easy to implement, will also allow the inclusion of other functionalities according to existing requirements, proving to be a feasible tool to deal with the problems discussed in the current article.

Lastly, it is expected that the catalogued data will allow identifying patterns and patients’ clinical profile as well as guiding the quality management processes of perioperative care.
